# Development of bicistronic plasmids and fusion proteins for clinical translation of tumor immune reprogramming

**DOI:** 10.1016/j.omta.2026.201708

**Published:** 2026-02-28

**Authors:** Joanna Yang, Sabrina S. Chen, Ethan Idnani, Sydney R. Shannon, Kathryn Luly, Charina S. Fabilane, D. Scott Wilson, Jamie B. Spangler, Stephany Y. Tzeng, Jordan J. Green

**Affiliations:** 1 Department of Biomedical Engineering, Johns Hopkins University School of Medicine, Baltimore, MD 21231, USA; 2 Translational ImmunoEngineering Center, Translational Tissue Engineering Center, Johns Hopkins University School of Medicine, Baltimore, MD 21231, USA; 3 Department of Chemical & Biomolecular Engineering, Johns Hopkins University, Baltimore, MD 21218, USA; 4 Department of Oncology and the Sidney Kimmel Comprehensive Cancer Center, Johns Hopkins University School of Medicine, Baltimore, MD 21231, USA; 5 Department of Ophthalmology, Johns Hopkins University School of Medicine, Baltimore, MD 21231, USA; 6 Department of Molecular Microbiology & Immunology, Johns Hopkins University School of Public Health, Baltimore, MD 21205, USA; 7 Department of Neurosurgery, Johns Hopkins University School of Medicine, Baltimore, MD 21205, USA; 8 Department of Materials Science & Engineering, Johns Hopkins University, Baltimore, MD 21218, USA; 9 Institute for NanoBioTechnology and the Bloomberg∼Kimmel Institute for Cancer Immunotherapy, Johns Hopkins University, Baltimore, MD 21231, USA

**Keywords:** gene therapy, gene delivery, non-viral, melanoma, immunotherapy, immunoengineering, T cell, plasmid, bioengineering, cancer

## Abstract

Delivery of 4-1BB ligand (4-1BBL) and interleukin 12 (IL-12) via poly(β-amino ester) (PBAE) nanoparticles (NPs) enables reprogramming of tumor cells into tumor-associated antigen-presenting cells (tAPCs), stimulating anti-tumor immune responses. Existing work on 4-1BBL/IL-12 NPs employs a dual-plasmid system and plasmid backbones containing antibiotic selection, both of which represent challenges for clinical translation. Production of two plasmids adds manufacturing complexity and costs, with amplification and purification processes required for both plasmids. Additionally, regulatory agencies discourage the use of antibiotic-resistance components in gene therapies due to concerns around rising antibiotic resistance. Here, we describe an approach to address manufacturing and regulatory challenges in translating 4-1BBL/IL-12 NPs into the clinic by engineering bicistronic plasmids co-expressing 4-1BBL and IL-12 via a T2A peptide or a (G_4_S)_5_ linker and utilizing smaller, antibiotic resistance-free nanoplasmid (NanoP) backbones. *In vivo,* we demonstrated bicistronic and NanoP-based tAPC NPs exhibited similar or improved survival compared to separate plasmid delivery of 4-1BBL and IL-12 in a B16-F10 tumor model. These results support the translation of bicistronic, antibiotic resistance gene-free plasmid strategies to advance tAPC PBAE-NPs and, more broadly, gene-delivered therapies toward the clinic.

## Graphical abstract

**Figure:**
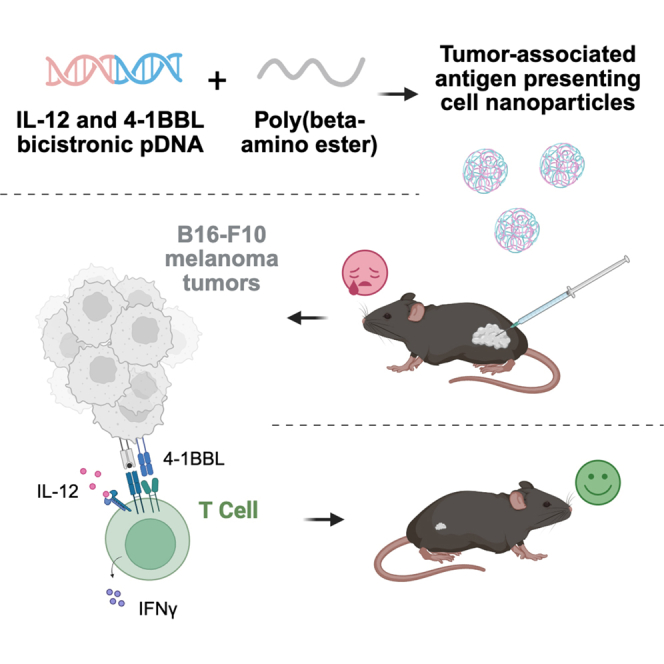

## Teaser

Bicistronic IL-12 and 4-1BBL plasmid DNA was delivered via Poly(beta-amino ester) nanoparticles into murine B16-F10 melanoma tumors. These immunomodulatory signals result in tumor reprogramming into tumor-associated antigen-presenting cells (tAPC) and stimulate an anti-tumor response. These bicistronic, antibiotic-free plasmids represent a crucial step in clinical translation for the tAPC platform.

## Introduction

T cell activation requires the coordination of three signals between T cells and antigen-presenting cells (APCs). Signal 1 consists of the T cell receptor interaction with a major histocompatibility complex (MHC): antigen complex, signal 2 encompasses co-stimulatory receptors engaging with their respective ligands, and signal 3 includes secreted cytokines. Prior research has demonstrated that poly(β-amino ester) (PBAE) nanoparticle (NP)-mediated co-delivery of plasmids encoding 4-1BB ligand (4-1BBL) (signal 2) and interleukin 12 (IL-12) (signal 3) can reprogram tumor cells into tumor-associated antigen-presenting cells (tAPCs) and generate an anti-tumor immune response.^1^ This approach has been validated in a variety of solid tumors, including melanoma, Merkel cell carcinoma,^2^ colorectal carcinoma, and breast cancer.^3^

However, translating gene therapy strategies into the clinic requires additional optimization of the manufacturing feasibility and safety profiles of genetic constructs. Current dual-signal delivery methods rely on co-delivery of two separate genetic constructs (i.e., one DNA plasmid encoding 4-1BBL and a second DNA plasmid encoding IL-12). Preparation of clinical-grade plasmid DNA requires amplification and purification for both plasmids, and a dual-plasmid system effectively doubles the DNA manufacturing cost compared to a single-plasmid system.^4^ A single-plasmid system additionally ensures expression of both genes in the same cell upon delivery. Furthermore, the presence of antibiotic resistance genes in plasmids may also elicit regulatory and safety concerns for clinical applications.^5^ To address these translational barriers, we developed several bicistronic versions of 4-1BBL and IL-12 so that both proteins are expressed from one plasmid.

We combined the two signals onto a single plasmid via two methods: a self-cleaving 2A^6^ sequence and a glycine-glycine-glycine-glycine-serine (G_4_S)_n_ peptide tether.^7^^,^^8^^,^^9^ In the 2A format, 4-1BBL and IL-12 are transcribed from a single plasmid but form two distinct proteins, while in the (G_4_S)_n_ format, the normally secreted IL-12 is tethered to the surface-bound 4-1BBL as a fusion protein.

2A peptides have been widely explored for the expression of recombinant proteins in a multi-cistronic manner, such as the four CD3 protein subunits to rescue T cell function.^10^ 2A peptides are also utilized to express both the heavy and light chains of an antibody *ex vivo* from the same plasmid.^11^^,^^12^^,^^13^ glycine-serine (GS) linkers have been widely explored in protein engineering as flexible linkers to connect protein domains.^14^ We chose (G_4_S)_n_, for the tethering of IL-12 to 4-1BBL^15^ as this linker has been validated extensively in both research and pharmaceutical applications.^16^^,^^17^^,^^18^ We hypothesized that using a linker to tether IL-12 to 4-1BBL offered two potential benefits: (1) ensuring IL-12 remains local to the tumor due to tethering to the cell surface and (2) enhancing T cell activation by increasing the effective concentration of 4-1BBL and IL-12 through their spatial proximity.^19^

Another barrier to clinical translation for many research-grade non-viral gene therapies, including the current tAPC approach, is the presence of antibiotic resistance genes in the plasmids. These genes pose a risk of horizontal transfer to bacterial populations in the human gut microbiome. Their removal from plasmid systems can help prevent the spread of antibiotic resistance, avoid potential allergic reactions in patients, and streamline compliance with good manufacturing process (GMP).^20^ The FDA also recommends against the usage of antibiotic selection markers in gene therapy.^21^ To eliminate the antibiotic resistance gene from the plasmid backbone, we employed the Nanoplasmid Vector System from Aldevron, which has already been utilized in several clinical trials.^22^

Taken together, the plasmid advancements we have implemented overcome critical barriers to the clinical translation of DNA tAPC NPs and other co-delivery-based therapeutic strategies by streamlining the manufacturing pipeline and enhancing the regulatory readiness of the platform. In this study, we optimized the 2A and GS linker bicistronic plasmid format for our therapeutic transgenes *in vitro*, evaluated the efficacy of these plasmids *in vivo*, and demonstrated the therapeutic efficacy of the optimized bicistronic plasmid in a nanoplasmid (NanoP) backbone *in vivo*. Coupled with NP-mediated administration, our bicistronic, antibiotic-free plasmid design strategy offers a potent and localized immunomodulatory effect with the potential for improved safety and translational feasibility of non-viral gene therapy approaches.

## Results

### Engineering bicistronic plasmids for co-expression of 4-1BBL and IL-12

**Figure 1:**
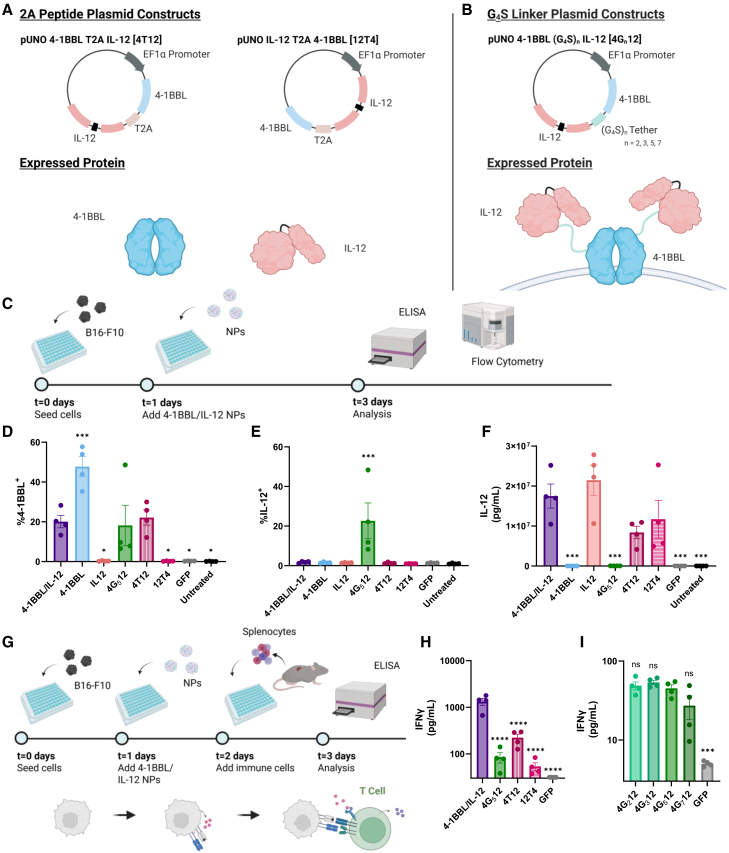
*In vitro* validation of delivery and function of bicistronic plasmids (A) Mouse *Tnfsf9* (4-1BBL) and mouse *IL12b-IL12a* (IL-12) were assembled onto a single plasmid separated with 2A peptide. (B) Mouse 4-1BBL and mouse IL-12 were assembled onto a single plasmid separated by a (G4S)n linker. (C) B16-F10 cells were transfected with bicistronic plasmids as shown in the experimental timeline. (D) 4-1BBL expression levels after co-delivery of 4-1BBL and IL-12 as separate plasmids or bicistronic plasmids as determined by flow cytometry (one-way ANOVA, Dunnett’s test, compared to 4-1BBL/IL-12 as separate plasmids). (E) Surface IL-12 expression after co-delivery of 4-1BBL and IL-12 as separate plasmids or bicistronic plasmids as determined by flow cytometry (one-way ANOVA, Dunnett’s test, compared to 4-1BBL/IL-12 as separate plasmids). (F) IL-12 expression in cell culture supernatant after co-delivery of 4-1BBL and IL-12 as separate plasmids or bicistronic plasmids as determined by enzyme-linked immunosorbent assay (ELISA) (one-way ANOVA, Dunnett’s test, compared to 4-1BBL/IL-12 as separate plasmids). (G) B16-F10 cells were reprogrammed and co-cultured with splenocytes. (H) Comparison of IFNγ expression in cell culture supernatant between plasmids as determined by ELISA (one-way ANOVA, Dunnett’s test, compared to 4-1BBL/IL-12 as separate plasmids). (I) Comparison of IFNγ expression between plasmids with differing lengths of the (G4S)n tether as determined by ELISA (one-way ANOVA, Dunnett’s test, compared to 4G_5_12). Significance is represented by ∗*p* ≤ 0.05, ∗∗*p* ≤ 0.01, ∗∗∗*p* ≤ 0.001, and ∗∗∗∗*p* ≤ 0.0001. Each data bar represents mean ± standard error of the mean (SEM) with four biological replicates.

We created six plasmid constructs containing 4-1BBL and IL-12, two of which incorporated the 2A peptide sequence, and four with varying lengths of the (G_4_S)_n_ peptide tether (Figures 1A and 1B; Table S1). T2A, derived from Thosea asigna virus, was chosen as the 2A sequence because it has the highest cleavage efficiency among the 2A peptides,^11^ thus ensuring that the proteins would not remain linked. In plasmids with the T2A sequence, one was formed with *Tnfsf9* (4-1BBL) at the N terminus (4T12), and the other was formed with *IL12b-IL12a* (IL-12) at the N terminus (12T4) to explore if sequence order affected expression and efficacy of tumor reprogramming. In the plasmids encoding the fusion protein with the (G_4_S)_n_ tether, four lengths of the (G_4_S)_n_ repeat (*n* = 2, 3, 5, and 7), corresponding to 10, 15, 25, and 35 amino acids were tested to evaluate which tether length resulted in the most effective tumor reprogramming. These constructs were labeled as 4G_n_12 and designed so the (G_4_S)_n_ repeat is positioned between the C terminus of *Tnfsf9* (4-1BBL) and the N terminus of *IL12b-IL12a* (IL-12) in order to enforce extracellular expression of IL-12, as 4-1BBL is a type 2 transmembrane protein with a C-terminal extracellular domain.^23^ All plasmids were cloned into the pUNO1 backbone.

### Optimized PBAE-NP formulation enhances *in vitro* transfection

B16-F10 melanoma cells were selected to test the transfection efficiency of our engineered bicistronic plasmids since the tAPC platform has previously been validated in this model.^1^ GFP plasmid delivery via seven PBAE-NPs was evaluated in order to select a formulation that enabled robust transfection and would facilitate downstream analysis of bicistronic plasmid protein expression. PBAE synthesis and structures can be found in Figures S1A–S1C. Each PBAE was evaluated at 30, 60, and 90 w/w (weight/weight polymer mass to DNA mass ratio) with 600 ng of DNA. The five formulations that achieved the highest transfection efficacy were PBAE 4-5-6 at 30 w/w (85 (1)%), PBAE 4-4-6 at 60 w/w (82 (2)%), PBAE 5-3-6 at 30 w/w (79 (1)%), PBAE 4-4-6 at 90 w/w (78 (1)%), and PBAE 4-5-39 at 60 w/w (70 (1)%) (Figure S1D). PBAE 4-4-6, also known as 2-(3-aminopropylamino)ethanol end-terminated poly(1,4-butanediol diacrylate-co-4-amino-1-butanol), constituted two of the five formulations with the highest transfection and was thus chosen for further *in vitro* testing. The 60 w/w formulation was selected due to higher GFP geometric mean fluorescence intensity (MFI) over untreated cells (∼460-fold) (Figure S1E) and higher cellular viability (Figure S1F) compared to the 90 w/w formulation.

### Bicistronic plasmids enable co-expression of 4-1BBL and IL-12 in multiple formats

The various plasmids we designed were assessed *in vitro* to compare 4-1BBL and IL-12 expression when the two immunomodulatory signals were delivered on a single plasmid or on separate plasmids (Figure 1C). Flow cytometry analysis of transfected cells showed that 20 (6)% of cells were 4-1BBL^+^ when 4-1BBL and IL-12 were delivered on separate plasmids (4-1BBL/IL-12) (Figure 1D). In the 4-1BBL^+^ only group, expression of 4-1BBL^+^ was significantly higher, as approximately twice the mass of 4-1BBL plasmid was delivered than in the group with 4-1BBL/IL-12. Notably, when compared to co-delivery of 4-1BBL and IL-12 individual plasmids, the percentage of 4-1BBL^+^ cells was not significantly different following transfection with 4G_5_12 (18 (10)%) or 4T12 (22 (4)%). However, the percentage of 4-1BBL^+^ cells was significantly lower in the 12T4 condition (0.2 (0.1)%) compared to co-delivery of the two plasmids (detailed statistical analysis is provided in Table S2). These results indicate that 4-1BBL can be delivered in a bicistronic format without negatively impacting levels of protein expression.

Flow cytometry staining for IL-12 revealed that 23 (9)% of cells that received 4G_5_12 expressed IL-12 on the cell surface (Figure 1E). Although IL-12 is naturally a secreted cytokine, these results indicate that IL-12 was successfully tethered, thus not secreted, to the C terminus of 4-1BBL via the GS linker. Additionally, this demonstrates the presence of IL-12 on the extracellular side of the cell.

ELISA measurement detected no significant differences in secreted IL-12 when the cytokine was delivered on a separate plasmid (18 (3) μg/mL) compared to delivery of 4T12 (8 (2) μg/mL) and 12T4 (12 (5) μg/mL) (Figure 1F). On the other hand, IL-12 concentration in the supernatant was significantly lower (0.030 (0.004) μg/mL) upon delivery of 4G_5_12. Paired with the detection of cell-surface IL-12 via flow cytometry, this result confirmed that IL-12 was successfully tethered to 4-1BBL in the context of this fusion protein. Fluorescence microscopy further verified the presence of IL-12 only on the cell surface in B16-F10 cells that were transfected with 4G_5_12 (Figure S2).

### tAPC delivery to cancer cells induces robust immune cell activation *in vitro*

After confirming the expression of 4-1BBL and IL-12 from the bicistronic plasmids, we assessed whether T cell activation was impacted by delivery of various plasmid formats. B16-F10 melanoma cells were treated with NPs delivering 4-1BBL and IL-12 as separate plasmids, 4G_5_12, 4T12, 12T4, or GFP, and then co-cultured with C57BL/6J splenocytes (Figure 1G). IFNγ secretion was quantified as a measure of immune stimulation typical of an anti-tumor response.

IFNγ expression was significantly higher when the plasmids were delivered separately (1,300 (200) pg/mL) compared to delivery of 4G_5_12 (80 (20) pg/mL), 4T12 (220 (40) pg/mL), or 12T4 (50 (10) pg/mL) (Figure 1H).

We then investigated the effects of modifying linker length in the 4-1BBL (G_4_S)_n_ IL-12 construct. Linker length represents the maximum distance the tethered IL-12 can be from the surface bound 4-1BBL and has little effect on the quantity of IL-12 present. If similar levels of IL-12 were expressed, then linker length would not be expected to affect IFNγ levels. However, if IL-12 were sterically hindered as a result of using shorter linkers, then IFNγ levels would be expected to be lower in groups with shorter linkers. Thus, multiple increasing linker lengths were tested. IFNγ levels were not significantly different among groups treated with constructs of different linker lengths although variability was greater for the construct with the longest linker length (G_4_S)_7_ (Figure 1I). Hence, the 4G_5_12 construct was used for subsequent studies.

### NP delivery of bicistronic 4-1BBL and IL-12 plasmids extends survival in B16-F10 tumor model

**Figure 2:**
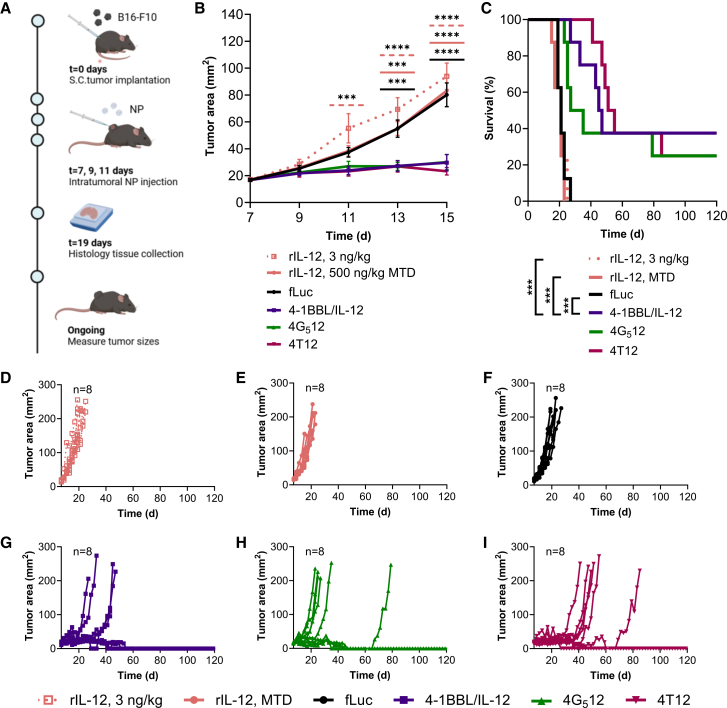
B16-F10 tumor-bearing mice survived longer after intratumoral injection of 4-1BBL- and IL-12-loaded NPs (A) Experimental timeline of mice inoculated with B16-F10 tumors is shown. (B) Growth curves of B16-F10 tumors treated with systemic recombinant IL-12 (rIL-12), luciferase (fLuc) NPs, 4-1BBL/IL-12 NPs, 4G_5_12 NPs, or 4T12 NPs (two-way ANOVA, Dunnett’s test, compared to 4-1BBL/IL-12-treated mice). Each data bar represents mean ± SEM with eight biological replicates. (C) Survival curves for mice treated with rIL-12, luciferase NPs, or tAPC NPs (log rank test with Bonferroni correction for multiple comparisons). (D–I) Tumor growth curves for each treatment group. Data points represent mean ± SEM with eight biological replicates. Significance is represented by ∗*p* ≤ 0.05, ∗∗*p* ≤ 0.01, ∗∗∗*p* ≤ 0.001, and ∗∗∗∗*p* ≤ 0.0001.

To determine whether *in vitro* immune cell activation by tAPCs would translate into anti-tumor activity *in vivo*, C57BL/6J mice were inoculated with B16-F10 melanoma cells (*t* = 0) and received intratumoral injections (*t* = 7, 9, and 11 days) of 2-(3-aminopropylamino)ethanol end-terminated poly(1,4-pentanediol diacrylate-co-3-amino-1propanol) PBAE NPs, or 5-3-6 PBAE NPs, delivering a control plasmid (luciferase), 4-1BBL, and IL-12 (as separate plasmids, denoted 4-1BBL/IL-12), 4G_5_12, or 4T12 (Figure 2A). About 50 μL of NP with a final DNA concentration of 0.2 mg/mL and a PBAE/DNA mass ratio of 30 w/w was injected per mouse. The PBAE 5-3-6 was employed in these studies as it had previously been optimized for delivery in mouse models. Additionally, we administered intravenous recombinant IL-12 (rIL-12) (*t* = 7, 11, 14, 18, 21, and 25 days), as this therapy has been previously evaluated in a phase 1 clinical trial in various cancer types, including melanoma.^24^^,^^25^ Two rIL-12 treatment cohorts were included, dosed at either the previously reported maximum tolerated dose (MTD) of IL-12 in patients (500 ng/kg), or the lowest reported dose (3 ng/kg). All mice received 100 μg of systemic anti-PD1 (*t* = 7, 9, and 11 days), as anti-PD1 therapy is a gold-standard immunotherapy for metastatic melanoma currently used in the clinic. This was therefore used as a benchmark in the studies in order to show that combination treatment with the NPs is beneficial. By delivering 4-1BBL and IL-12 into tumor cells, T cell activation against the tumor cells was increased, and this is expected to synergize with anti-PD1, which blocks the inactivation of T cells.^26^

Mice that received a systemic injection of rIL-12 or intratumoral luciferase NPs experienced significantly faster tumor growth compared to those that had received DNA NPs carrying 4-1BBL and IL-12 in any of the plasmid formats (Figure 2B). In comparison, there was no significant difference between mice receiving 4-1BBL/IL-12 NPs and either 4G_5_12 NPs or 4T12 NPs. Similarly, mice receiving 3 ng/kg of IL-12 and 500 ng/kg rIL-12, both exhibited a median survival of 21 days, and mice receiving luciferase NPs also exhibited an average survival of 21 days (Figure 2C). Moreover, there were no long-term survivors in the rIL-12- or luciferase NP-treated groups. Mice that received 4-1BBL/IL-12 (median survival of 46 days and 3 long-term survivors), 4T12 (median survival of 53 days and 2 long-term survivors), and 4G_5_12 NPs (median survival of 31 days and 2 long-term survivors) showed considerably better outcomes. Furthermore, no significant difference in survival was observed between mice that received 4-1BBL/IL-12 NPs and mice that received 4G_5_12 NPs or 4T12 NPs (Figure 2C). Individual mouse tumor plots highlighted the delay in tumor growth for mice treated with gene-delivered 4-1BBL and IL-12 compared to the rIL-12 and luciferase NP cohorts (Figures 2D–2I).

Dynamic light scattering (DLS) analysis revealed that PBAE-NPs used for *in vivo* studies were in the size range of 200 nm in hydrodynamic diameter, with slightly negative zeta potentials. NP diameters were measured for co-delivered 4-1BBL/IL-12 NPs (199 (5) nm), 4G_5_12 NPs (205 (2) nm), 4T12 NPs (204 (3) nm), and luciferase NPs (215 (6) nm) (Figure S3A). The polydispersity indices were all below 0.15, indicating a narrow size distribution (Figure S3B). Zeta potentials were also measured for co-delivered 4-1BBL/IL-12 NPs (−9 (3) mV), 4G_5_12 (−4.5 (3) mV), 4T12 (−13 (1) mV), and luciferase (−5 (1) mV) (Figure S3C). No statistically significant differences were detected among the 4-1BBL/IL-12 NP sizes, polydispersity indices, or zeta potentials compared to either of the bicistronic plasmids.

Transmission electron microscopy (TEM) was employed to visualize dried NPs formed with separate 4-1BBL and IL-12 plasmids (Figure S3D) and NPs formed with the bicistronic 4T12 plasmid (Figure S3E) that were used *in vivo*. Spherical shapes without aggregation were observed, confirming the successful formation of distinct NPs.

### NP delivery of bicistronic 4-1BBL and IL-12 plasmids leads to CD4^+^ and CD8^+^ T cell infiltration into tumor

**Figure 3:**
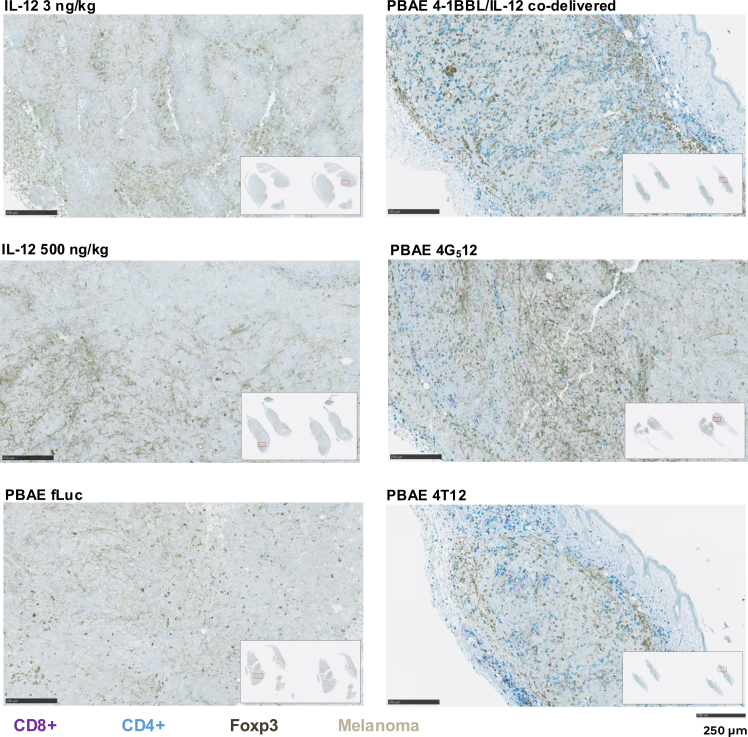
Immunohistochemistry staining of harvested B16-F10 tumors reveals immune infiltration Tumor tissue excised from mice treated systemically with 3 ng/kg or 500 ng/kg of recombinant IL-12 or intratumorally with luciferase NPs, co-delivered 4-1BBL and IL12 plasmid NPs, 4G_5_12 NPs or 4T12 NPs. Melanin appears as beige brown. CD8^+^ cells are stained purple, CD4^+^ cells teal, and FOXP3^+^ cells dark brown. Scale bars, 250 μm.

Tumors were excised 1 day after the last injection of rIL-12 or NPs and stained for CD8^+^ (purple), CD4^+^ (teal), and FOXP3^+^ (dark brown) T cells. Melanoma can be visualized as the beige/light brown regions due to melanin deposits (Figure 3). Tumors in mice that had received rIL-12 or luciferase NPs exhibit little to no T cell infiltration. Tumors in mice that received 4-1BBL/IL-12 NPs demonstrated high levels of CD4^+^ and CD8^+^ T cell infiltration into the tumor. Tumors treated with 4G_5_12 NPs showed similar CD4^+^ T cell infiltration compared to the 4-1BBL/IL-12 group, although fewer CD8^+^ T cells were observed. This observation raises the question of whether tethering IL-12 to the cell surface might limit effective immune cell recruitment into solid tumors and likely explains the reduced therapeutic efficacy compared to other plasmid formats. Tumors in the 4T12 group had similar levels of CD4^+^ and CD8^+^ infiltration compared to those in the 4-1BBL/IL-12 group. FOXP3^+^ Treg infiltration did not seem to vary between treatment groups, indicating that there was a lack of an anti-inflammatory response.

### tAPC NP delivery can elicit long-lasting anti-tumor immune responses

**Figure 4:**
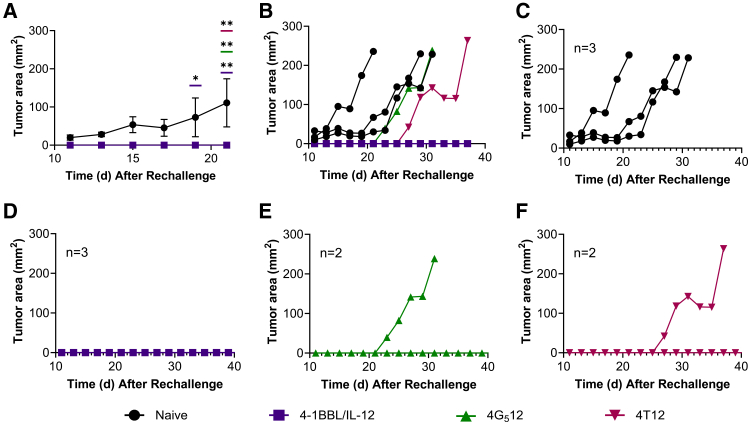
Tumor rechallenge to assess durability of tAPC NP-induced anti-tumor effects (A) Long-term survivors were rechallenged with B16-F10 tumors on day 100 alongside age-matched naive mice (two-way ANOVA, Dunnett’s test, compared to naive mice). (B) Average size of new tumors in naive mice compared to 4-1BBL/IL-12-, 4G_5_12-, or 4T12-treated mice. Individual tumor growth in (C) naive mice (*n* = 3), (D) 4-1BBL/IL-12 NP-treated mice (*n* = 3), (E) 4G_5_12 NP-treated mice (*n* = 2), and (F) 4T12 NP-treated mice (*n* = 2). Data points represent mean ± SEM. Significance is represented by ∗*p* ≤ 0.05, ∗∗*p* ≤ 0.01, ∗∗∗*p* ≤ 0.001, and ∗∗∗∗*p* ≤ 0.0001.

One hundred days after the initial tumor implantation, seven long-term survivors and three age-matched naive mice were inoculated with B16-F10 tumors on the opposite flank. Three long-term survivors belonged to the group treated with 4-1BBL/IL-12, two to the group treated with 4G_5_12, and two to the group treated with 4T12. All naive mice developed tumors with a median survival of 25 days. Tumor growth in naive mice was also significantly faster than in mice that had been previously treated (*p* < 0.0001) (Figure 4A). In groups that had been previously treated, if a new tumor formed, it grew later than in the previously untreated naive mice (Figure 4B). All three naive mice developed tumors, no mice treated with 4-1BBL/IL-12 NPs developed tumors, and one of the two mice developed a tumor in the groups treated with 4G_5_12 or 4T12 (Figures 4C–4F). In a prior study in our lab,^1^ mice previously treated with 4-1BBL/IL-12 NPs had higher B16-F10 antigen-specific T cells and delayed tumor growth. The resistance of new tumor growth in 4G_5_12 or 4T12 NP-treated mice demonstrates that the long-lasting anti-tumor immune response is not specific to the two-plasmid format.

### Translationally relevant antibiotic resistance gene-free NanoP demonstrate similar efficacy to pUNO1 plasmids

**Figure 5:**
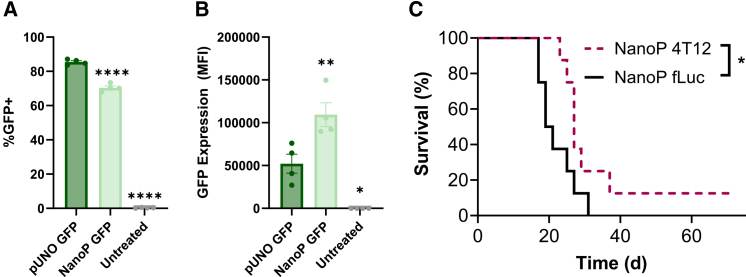
Survival in B16-F10 mouse model with antibiotic-gene free NanoP (A) Transfection of B16-F10 cells using 600 ng of pUNO1 or nanoplasmid (NanoP) backbones as determined by flow cytometry. (B) GFP expression, determined by flow cytometry. Each data bar represents mean ± SEM with four biological replicates. (C) Average tumor growth of tumors treated with nanoplasmid 4T12 NPs + anti-PD1 vs. nanoplasmid luciferase NPs + anti-PD1. Each data curve represents eight biological replicates. Significance is represented by ∗*p* ≤ 0.05, ∗∗*p* ≤ 0.01, ∗∗∗*p* ≤ 0.001, and ∗∗∗∗*p* ≤ 0.0001.

To ensure compliance with GMP production, we utilized nanoplasmids (NanoPs) from Aldevron to create an antibiotic resistance-free version of the 4T12 bicistronic plasmid, a minimal plasmid without extra bacterial elements. GFP versions of the NanoP and pUNO1 plasmids were delivered to B16-F10 cells to compare expression. NPs were formulated with 600 ng of plasmid and 60 w/w of 4-4-6 PBAE in 20 μL. NanoP NPs demonstrated 70 (2)% transfection efficiency, while pUNO1 NPs demonstrated 85 (1)% efficiency (Figure 5A). However, GFP MFI with NanoP NPs was more than 2-fold greater than with pUNO1 NPs (Figure 5B). Percent GFP is a binary measurement, representing whether cells expressed the delivered GFP gene, while MFI represents the strength of the GFP signal, a proxy measurement of the amount of protein produced. In this context, transfection with NanoP resulted in much stronger GFP signal in slightly fewer cells, which potentially suggests stronger downstream expression of T cell activation signals 4-1BBL and IL-12. This may be due to the smaller size of the NanoP compared to the pUNO1 plasmid, allowing for higher copy number of the delivered gene by mass.

C57BL/6J mice were inoculated with B16-F10 melanoma cells on *t* = 0 and treated intratumorally (*t* = 9, 11, 16, and 18 days) with 5-3-6 PBAE-NPs co-delivering plasmids encoding 4-1BBL/IL-12 separately using either the pUNO1 or NanoP backbone vectors. Tumors that were treated with luciferase NPs grew faster than those treated with tAPC NPs, regardless of plasmid used (Figures S4A and S4B). Mice that were treated with luciferase NPs demonstrated a median survival time of 20 days, which increased to 22 days with the additional administration of anti-PD1 (Figures S4C and S4D). Mice treated with tAPC NPs formed with pUNO1 plasmids demonstrated a median survival time of 29 days, increasing to 32 days with anti-PD1, and mice treated with tAPC NPs formed with NanoPs demonstrated a median survival time of 32 days, increasing to 35 days with addition of anti-PD1. Only the group treated with NanoP tAPC NPs and anti-PD1 had a long-term survivor.

In a further study, NanoP luciferase NPs were compared to NanoP bicistronic 4T12 NPs (Figure 5C). The group treated with NanoP luciferase yielded no long-term survivors, while the group treated with 4T12 NPs yielded one long-term survivor. Median survival was 20 days in the luciferase group and 27 days in the 4T12 group. Survival was significantly better in the 4T12 group (∗*p* = 0.0358). Average tumor growth and individual tumor growth can be found in Figures S5A and S5B. Mice treated with NanoP luciferase NPs exhibited significantly larger tumors compared to those treated with NanoP 4T12 NPs by day 15 (Figure S5A). In mice receiving NanoP 4T12 NPs, tumors reached euthanasia criteria later than those receiving NanoP luciferase NPs, as reflected in the rightward shift in individual tumor sizes (Figure S5B).

## Discussion

Despite increased immunotherapy approvals in recent years, many solid tumors remain difficult to treat due to a lack of effective antigen presentation and T cell activation within the tumor.^27^ Local expression of 4-1BBL and IL-12 can reprogram tumor cells into tAPCs and effectively drive anti-tumor response.^1^ However, clinical translation of this strategy faces manufacturing and regulatory challenges. To address these barriers, we took a two-pronged approach. First, we engineered bicistronic constructs that enable co-expression of both immune-modulating genes from a single plasmid, simplifying delivery and reducing manufacturing costs. Second, we demonstrated that removal of the traditional antibiotic resistance gene, which will be important for regulatory and manufacturing concerns, does not negatively impact therapeutic efficacy. These findings support eventual translation of the tAPC NPs into clinical trials.

IL-12 has been widely explored as a treatment for its anti-cancer effects, as it activates T cells and natural killer cells and enhances the immune system’s ability to recognize and eliminate tumor cells.^25^ For this study, two doses were tested: 3 ng/kg and 500 ng/kg, the latter of which was the MTD observed in humans. While other preclinical studies of rIL-12 in B16-F10 models demonstrated varying anti-tumor efficacy of systemically delivered rIL-12 in the same model, these studies administered much higher doses than the MTD in humans and would be difficult to translate into the clinic.^28^^,^^29^ No anti-tumor effect was observed for either rIL-12 dose in our study, which was unsurprising given the relatively low dosage and emphasizes the difficulty of translating rIL-12 to the clinic with its very small therapeutic window.

Clinically, rIL-12 has been delivered both alone as a recombinant protein or as a gene therapy, as well as synergistically with chemotherapy, radiotherapy, and other immunotherapies, such as cancer vaccines and checkpoint inhibitors.^30^^,^^31^ However, despite its potential, IL-12 in any form has not been approved for cancer treatment, due to dose-limiting toxicity resulting from high systemic levels of pro-inflammatory cytokines, otherwise known as cytokine release syndrome.^31^ Several alternative strategies to confine systemic delivery of IL-12 to the local tumor environment have been investigated preclinically, both with rIL-12 protein (rIL-12) and IL-12 gene therapies. Approaches in rIL-12 delivery include using tumor-protease-cleavable linkers or tumor pH-mediated activation to localize IL-12 activity to the tumor, while approaches for IL-12 gene delivery include using mRNA linker locks, where mRNA expression only occurs in the matrix metalloproteinase-2 (MMP-2) rich tumor domain.^32^^,^^33^^,^^34^

Prior studies have also demonstrated that confinement of IL-12 to the tumor microenvironment via an intratumoral injection may limit systemic toxicities.^35^ We elected to connect IL-12 to 4-1BBL via a flexible (G_4_S)_5_ linker, which is commonly used in protein engineering and design. Paired with an intratumoral injection, we hypothesized that tethering IL-12 to a surface protein (4-1BBL) would result in IL-12 confinement to the tumor, potentially providing two advantages: (1) to reduce systemic availability resulting from secreted IL-12 exiting the tumor microenvironment after intratumoral injection and (2) to potentially increase T cell activation due to the spatial proximity of signal 2 and signal 3. Altering the length of the GS tether did not appear to influence IFNγ production *in vitro*, although the longest linker length tested exhibited greater variance and slightly (though not statistically significant) lower levels of IFNγ. While we observed a decrease in secreted IL-12, the tethered construct ultimately did not result in a greater anti-tumor response compared to the co-delivered 4-1BBL and IL-12 formulations *in vivo* or *in vitro*. However, there may be potential to employ a tethered 4-1BBL and IL-12 to strengthen T cell responses in applications that utilize other strategies for local IL-12 administration.^36^^,^^37^

Bicistronic plasmids that have been engineered with 2A peptides result in the expression of two distinct proteins in approximately equimolar ratios.^38^ The ribosome skipping results in most of the 2A domain remaining on the C terminus of the protein that is expressed first, while a single proline scar remains on the N terminus of the protein expressed second. Due to the short sequence of the 2A peptide, it has been reported that protein functions are not significantly impacted.^39^ However, in our *in vitro* studies characterizing the expression of 4-1BBL and IL-12, it appeared that expression of 4-1BBL following the T2A domain was significantly lower than when 4-1BBL was located before the T2A domain. Additionally, the T2A sequence was selected because it had been previously reported to have the highest cleavage efficiency, with some studies demonstrating ∼100% cleavage.^40^^,^^41^ Cleavage efficiency was not evaluated in this study but may vary when expressing different proteins, and analyzing this parameter in the future may be helpful in optimizing therapeutic efficacy.

In this study, we demonstrated that bicistronic plasmids resulted in significantly improved survival compared to luciferase NPs, as well as systemic IL-12 delivery, without losing therapeutic efficacy compared to co-delivered 4-1BBL/IL-12 plasmids. Interestingly, while IFNγ levels were significantly higher *in vitro* for 4-1BBL and IL-12 delivered as separate plasmids compared to the 4T12 or 12T4 constructs, this difference did not translate to lower efficacy *in vivo*. The median survival time in mice treated with 4T12 was the longest, at 53 days following a dosing regimen of three injections. PBAEs offer the advantage of being both biodegradable and biocompatible, which enables multiple administrations without observed toxic or immunogenic side effects.^42^^,^^43^ Administering additional injections could potentially extend the survival time even further. Moreover, tumor rechallenge demonstrated slower formation of a new tumor compared to previously untreated mice, reflecting the establishment of long-term anti-tumor response in some animals. The tAPC antigen-agnostic reprogramming approach used here can lead to cellular immune responses against varied tumor antigens, reducing the likelihood of tumor recurrence.^1^ Although NPs were not injected past the initial three doses, including after tumor rechallenge, additional PBAE-NP administrations may be performed to maintain durability if needed for a clinical application.^44^ If a patient experiences tumor recurrence, the NPs could be redosed into tumors to broaden the immunological reprogramming and increase the pool of T cells to aid in tumor regression. The PBAE synthetic polymers used are hydrolytically degradable in water and thus are anticipated to be minimally immunogenic, which broadens the potential therapeutic window to enable repeated dosing. This in contrast to certain other polymers used in NP delivery, such as polyethylene glycol (PEG), which is not easily degradable, used in mRNA-lipid nanoparticle (LNP) formulations, and has shown the potential for the development of anti-PEG antibodies, which can reduce the potency of subsequent dosing.^45^ Together, these studies demonstrate that the bicistronic 4T12 plasmid is a viable construct for future clinical translation.

Although 4-4-6 NPs were used for *in vitro* validation of plasmids, we used 5-3-6 NPs *in vivo*, as this formulation had been previously validated for intratumoral delivery in mouse models.^46^ Characterization of the NPs demonstrated that all NPs were ∼200 nm in diameter and displayed a negative zeta potential, indicating that the therapeutic effects from NP groups are not due to differences in biophysical properties of the NPs. Previous studies have indicated that the negative charge of the particles may also help with solid tumor delivery *in vivo*.^47^ The tumor extracellular matrix (ECM) is generally negatively charged, and delivery of a negatively charged NP may reduce NP interactions with the ECM, leading to deeper tumor penetration.^48^

Another gene therapy safety concern pertains to the risk of transferring antibiotic resistance genes to bacteria in the human microbiome. Antibiotic resistance represents a growing global health concern,^49^ making it an important regulatory consideration in gene therapies.^50^ Research-grade plasmids often contain an antibiotic resistance gene as a selection marker. However, in the context of the human microbiome, these plasmids may undergo horizontal gene transfer of their antibiotic resistance genes. As a result, the use of antibiotic resistance genes is discouraged by regulatory agencies. Here, new plasmid vectors without antibiotic selection markers were developed to comply with regulatory requirements.^20^ We employed the NanoP vector from Aldevron for delivery of 4-1BBL and IL-12 and evaluated these compared to the pUNO1 plasmids *in vivo*. We found that the NanoP lead to similar response rates compared to pUNO1 plasmids, and NanoP may even have a therapeutic advantage due to improved delivery efficiency. The improved therapeutic efficacy may be attributed to the smaller size of the NanoP compared to the pUNO1 plasmid, enabling a higher copy number of the transgene to be delivered on a per mass basis. Future work entails testing bicistronic NanoP constructs encoding 4T12.

Together, the improvements outlined in these studies align with current GMP and regulatory standards and lay the groundwork for eventual translation into clinical trials. Safe and effective non-viral reprogramming of the tumor immune microenvironment has the potential to induce endogenous cellular immune responses to treat both local solid tumors and systemic metastatic disease.

## Materials and methods

### Plasmid design

The plasmids in this study were created using the pUNO1 backbone containing a blasticydin antibiotic resistance gene, following standard cloning methods. Restriction enzymes NheI (New England Biolabs [NE Biolabs]; NEB, catalog no. R3131S) and SalI (NEB, catalog no. R0138S) were used to remove the inserts from Clonal Genes (Twist Biosciences) encoding mouse *Tnfsf9* (4-1BBL) (residues 1–309 in 4G_5_12, 4T12, and residues 566–875 in 12T4) and mouse *IL12b-IL12a* (IL-12) (P40:P35) (residues 335–851 in 4G_5_12, residues 342–880, and residues 1–538 in 12T4) with the linkers (residues 310–334) and T2A sequences (residues 310–333 in 4T12 and 539–559 in 12T4) noted in Figure 1. A table of the coding region for all plasmids is provided (see Table S1). In tandem, the pUNO1 backbone from the plasmid pUNO1 MCS (InvivoGen, catalog no. pUNO1-mcs) was digested with NheI and SalI. Ligation was performed using T4 DNA ligase (NEB, M0202S). Sequences of plasmids were verified via Sanger sequencing.

### Polymer synthesis

PBAEs were synthesized based on previously published protocols.^51^^,^^52^^,^^53^^,^^54^ Briefly, seven PBAEs were synthesized by Michael addition reactions: 4-4-6, 4-4-27, 4-5-6, 4-5-7, 4-5-27, 4-5-39, and 5-3-6, with these numerical codes denoting the polymer backbones, sidechains, and end caps in that order (Table 1).^55^ Backbone monomers (B4 and B5) were mixed neat with sidechain monomers (S3, S4, and S5) at 85°C overnight at a 1.1:1 backbone to sidechain ratio. Endcap monomers (E6, E7, E27, and E39) were combined with the formed polymer in tetrahydrofuran (THF). The resulting polymer was then washed in diethyl ether and stored in DMSO.

**Table 1:** Listing of the chemical monomers used to synthesize the poly(beta-amino ester)s

Polymer component	Monomer	Chemical name	Manufacturer	Catalog no.
Backbone	B4	1, 4-butanediol diacrylate	Alfa Aesar	1070-70-8
	B5	1,4-pentanediol diacrylate	Monomer-Polymer and Dajac Labs	36840-85-4
Sidechain	S3	3-amino-1propanol	Alfa Aesar	156-87-6
	S4	4-amino-1butanol	Thermo Fisher Scientific	13325-10-05
	S5	5-amino-1pentanol	Alfa Aesar	2508-29-4
Endcap	E6	2-(3-aminopropylamino)ethanol	Sigma Aldrich	4461-39-6
	E7	1-(3-aminopropyl)-4-methylpeperazine	Alfa Aesar	4572–031
	E27	4,7,10-trioxa-1,13-tridecanediamine	Sigma Aldrich	4246-51-9
	E39	1-(2-aminoethyl)piperazine	Alfa Aesar	140-31-8

The table lists the chemical monomers used for synthesis, their abbreviated names, and the manufacturer and catalog numbers for ordering.

### Cells

B16-F10 mouse melanoma cells were obtained from ATCC (ATCC, catalog no. CRL-6475). Cells were cultured in DMEM (Thermo Fisher Scientific, catalog no. 11965092) supplemented with 10% fetal bovine serum (Milliporesigma, catalog no. F4135-500ML) and 1% penicillin/streptomycin (Thermo Fisher Scientific, catalog no. 15140122) at 37°C and a humidified environment.

### *In vitro* NP formulation and transfection

B16-F10 cells were seeded at a cell density of 10,000 cells per well on day 0. On day 1, PBAEs and DNA were diluted in 25 mM sodium acetate, mixed, and self-assembled within minutes to form PBAE-NPs. In total, 600 ng of DNA were delivered to B16-F10 melanoma cells at 30, 60, and 90 w/w (weight/weight polymer mass to DNA mass ratio) in 20 μL of NPs. After 2 h, all media was changed. On day 2, cell viability was measured via 3-(4,5-dimethylthiazol-2-yl)-5-(3-carboxymethoxyphenyl)-2-(4-sulfophenyl)-2H-tetrazolium (MTS) assay (see viability: MTS assay), and on day 3, cells or culture supernatant were harvested for ELISA or flow cytometry for further analysis. In the NanoP comparison transfection, 600 ng of either pUNO1 GFP or NanoP GFP were delivered at 60 w/w in 20 μL of NPs. All other steps remained the same. NP dose is defined as the amount of DNA dosed into cells.

### Viability: MTS assay

After 24 h of cell transfection, cell media in each well was removed and replaced with a 100 μL of a 1:10 ratio of MTS assay buffer (Promega, catalog no. G3582) to cell media. The plate was incubated at 37°C for 1 h, and absorbance was measured at 490 nm.

### 4-1BBL and IL-12 expression detection

Cells were washed with 200 μL of PBS and detached from the plate using 30 μL of Trypsin-EDTA (Thermo Fischer Scientific, catalog no. 25300054). The cells were then quenched with 170 μL of fluorescence-activated cell sorting (FACS) buffer (PBS +10% fetal bovine serum [FBS]) and transferred to a v-bottom plate to be centrifuged at 300 rcf for 5 min at 4°C. The supernatant was removed, and cells were resuspended in 100 μL of FACS buffer. Cells were stained with antibodies against 4-1BBL and IL-12 (Table 2). Cells stained with IL-12 were imaged on the Zeiss AxioObserver fluorescence microscope. Each image was taken with the same exposure time and identically processed in the AxioVision software. For mouse IL-12 detection, cell supernatant was collected 48 h after cell transfection and measured via ELISA (BioLegend, catalog no. 433604).

**Table 2:** Listing of the antibodies used for staining

Fluorophore	Target	Clone	Manufacturer	Catalog no.	Dilution
PE	anti-mouse 4-1BB ligand	5F4	BioLegend	107105	80
Alexa Fluor	anti-mouse IL-12/IL-23 p40	C15.6	BioLegend	505214	100

The table lists the fluorophore, target, clone, manufacturer, catalog number, and dilution ratio for each antibody.

### Co-culture for IFNγ detection

For mouse IFNγ detection, mouse splenocytes were harvested from the spleen of one C57BL/6 mouse. The spleen was pressed through a 70-μm filter, incubated for 1 min with ACK buffer (Quality Biological, catalog no. 118-156-721) for red blood cell lysis, diluted in PBS, and passed through a 40-μm filter. Cells were centrifuged, resuspended in complete DMEM and 200,000 splenocytes co-cultured with B16-F10 cells 24 h after transfection. Cell supernatant was harvested 24 h after co-culture and measured via ELISA (BioLegend, catalog no. 430804) according to the manufacturer’s instructions.

### *In vivo* NP sizing and characterization

*In vivo* NPs were prepared with 0.2 mg/mL DNA in 75 μL, at 30 w/w using PBAE 5-3-6. After self-assembly, NPs were diluted 1:100 in 0.1× PBS and size and surface charge (zeta potential) were measured using a Malvern ZetaSizer Pro. For each NP formulation, *n* = 4 replicates were assessed for both size and zeta potential. A different NP was used in the *in vivo* studies than in the *in vitro* studies because the PBAE 5-3-6 had been previously optimized for intratumoral delivery in mouse models.

### TEM imaging

5-3-6 NPs for *in vivo* use were formulated in 25 mM sodium acetate and diluted in deionized water to 0.2 mg/mL DNA. Samples were transferred onto a carbon film 400 mesh copper grid (Electron Microscopy Sciences; Hatfield, PA, USA) and dried at room temperature for 5–6 h. Grids were washed with deionized water to remove excess salts, followed by staining in 1% Uranyl Acetate solution (Electron Microscopy Sciences; Hatfield, PA, USA). The grids were then dried overnight, and samples were imaged on the Hitachi 7600 TEM (Hitachi High-Tech; Tokyo, Japan).

### Survival study and monitoring

All animal studies were performed within the guidelines of the Johns Hopkins Animal Care and Use Committee under approved protocol numbers MO23E357 and MO24M284. A total of 3 × 10^5^ B16-F10 cells were implanted subcutaneously into the flanks of seven groups of C57BL/6J mice (*t* = 0). The groups were as follows: (1) PBAE-NPs delivering 4-1BBL and IL-12 plasmids (separate plasmids), (2) PBAE-NPs delivering 4T12, (3) PBAE-NPs delivering 12T4, (4) PBAE-NPs delivering 4G_5_12, (5) PBAE-NPs delivering luciferase plasmid, (6) maximum tolerated dose (MTD) of IL-12 protein, and (7) a low dose (3 ng/kg) of IL-12 protein. For groups receiving NPs, PBAE 5-3-6 and plasmid were diluted in 25 mM magnesium acetate (Sigma Aldrich, catolog no. 63052-100ML) and combined for a final DNA concentration of 0.2 mg/mL and a PBAE/DNA mass ratio of 30 w/w. RIL-12 protein (Thermo Fisher Scientific, catolog no. 210-12-10UG) was diluted in PBS. About 50 μL (10 μg DNA) of PBAE-NPs were injected intratumorally (*t* = 7, 9, and 11 in Figure 2; Figure 5 studies, *t* = 9, 11, 16, and 18 in Figure S4 study). IL-12 protein groups were delivered on *t* = 7, 11, 14, 18, 21, and 25 intravenously by either retro-orbital or tail vein injection. This IL-12 dosing strategy was selected to be delivered systemically and twice weekly based on previous studies reported in literature.^24^^,^^56^ All groups received 100 μg of systemic anti-PD1 (*t* = 7, 9,11 in Figure 2; Figure 5 studies, *t* = 9, 11, 16, 18 in Figure S4 study) intraperitoneally. Every 2 days, tumor area was measured as tumor length × width. Mice were euthanized when the area of the tumor exceeded 200 mm^2^ or for moribund states.

### Tumor rechallenge

In the survival study comparing different bicistronic plasmids, seven mice exhibited regressed tumors. These mice were rechallenged with 3 × 10^5^ B16-F10 cells on the left flank, opposite from the initial study. Age matched C57BL/6J mice were similarly implanted with B16-F10 cells. Every 2 days, surface area was measured as described above, and mice were euthanized when the tumor exceeded 200 mm^2^ or for moribund states.

## Data and code availability

All data used in this study are available within the article and its supplemental information. Materials are available upon request from the corresponding authors.

## Acknowledgments

Figures 1, 2A, 2E, 4A, and 6A were created with BioRender.com. The authors would like to thank the 10.13039/100000002NIH (R01CA228133 and P41EB028239 [to J.J.G.], R37CA246699 [to S.Y.T.], R01 EB029455 [to J.B.S.], and P41EB024495 [to J.J.G.]); the NSF (2143160 to J.B.S.); the 10.13039/100000005DOD (HT942523C0005 to J.B.S.) as well as the Goldhirsh-Yellin Foundation (to J.J.G.) and the 10.13039/100009858Gabrielle's Angel Foundation for Cancer Research (to J.B.S.) for support of this study. The Johns Hopkins Oncology Tissue Services core is funded by the 10.13039/100000002National Institutes of Health (P30CA006973).

## Author contributions

Conceptualization, J.Y., J.B.S., S.Y.T., and J.J.G.; data curation, formal analysis, and writing – original draft, J.Y.; funding acquisition, J.B.S., S.Y.T., and J.J.G.; investigation, J.Y., S.S.C., E.I., S.R.S., K.L., C.S.F., and S.Y.T.; methodology and validation, J.Y. and S.Y.T.; project administration, J.Y., S.Y.T., and J.J.G.; resources and supervision, S.Y.T. and J.J.G.; visualization, J.Y., S.S.C., S.Y.T., J.B.S., and J.J.G.; and writing – review and editing, J.Y., S.S.C., K.L., C.S.F., D.S.W., J.B.S., S.Y.T., and J.J.G.

## Declaration of interests

Johns Hopkins filed patents related to the technology discussed in the article with J.J.G., S.Y.T., J.B.S., and J.Y. as co-inventors. J.J.G. is also a board member, CSO, and co-founder of Cove Therapeutics; a manager, CTO, and co-founder of Dome Therapeutics; and a manager and co-founder of OncoSwitch Therapeutics. S.Y.T. is a manager and co-founder of OncoSwitch Therapeutics. Any potential conflicts of interest are managed by the Johns Hopkins University Committee on Outside Interests.

## Supplemental information

Document S1. Figures S1–S5 and Table S1

Table S2. Detailed statistical analysis

Document S2. Article plus supplemental information
