# Supplementary material for: Development of bicistronic plasmids and fusion proteins for clinical translation of tumor immune reprogramming
Source: Mol Ther Adv. 2026 Feb 28;34(2):201708. doi: 10.1016/j.omta.2026.201708 (PMC13148935; doi:10.1016/j.omta.2026.201708)
Supplement: Document S1. Figures S1–S5 and Table S1 [file mmc1.pdf]

OMTA, Volume *34*

Supplemental information

Development of bicistronic plasmids and fusion proteins for clinical translation of tumor immune reprogramming

Joanna Yang, Sabrina S. Chen, Ethan Idnani, Sydney R. Shannon, Kathryn Luly, Charina S. Fabilane, D. Scott Wilson, Jamie B. Spangler, Stephany Y. Tzeng, and Jordan J. Green

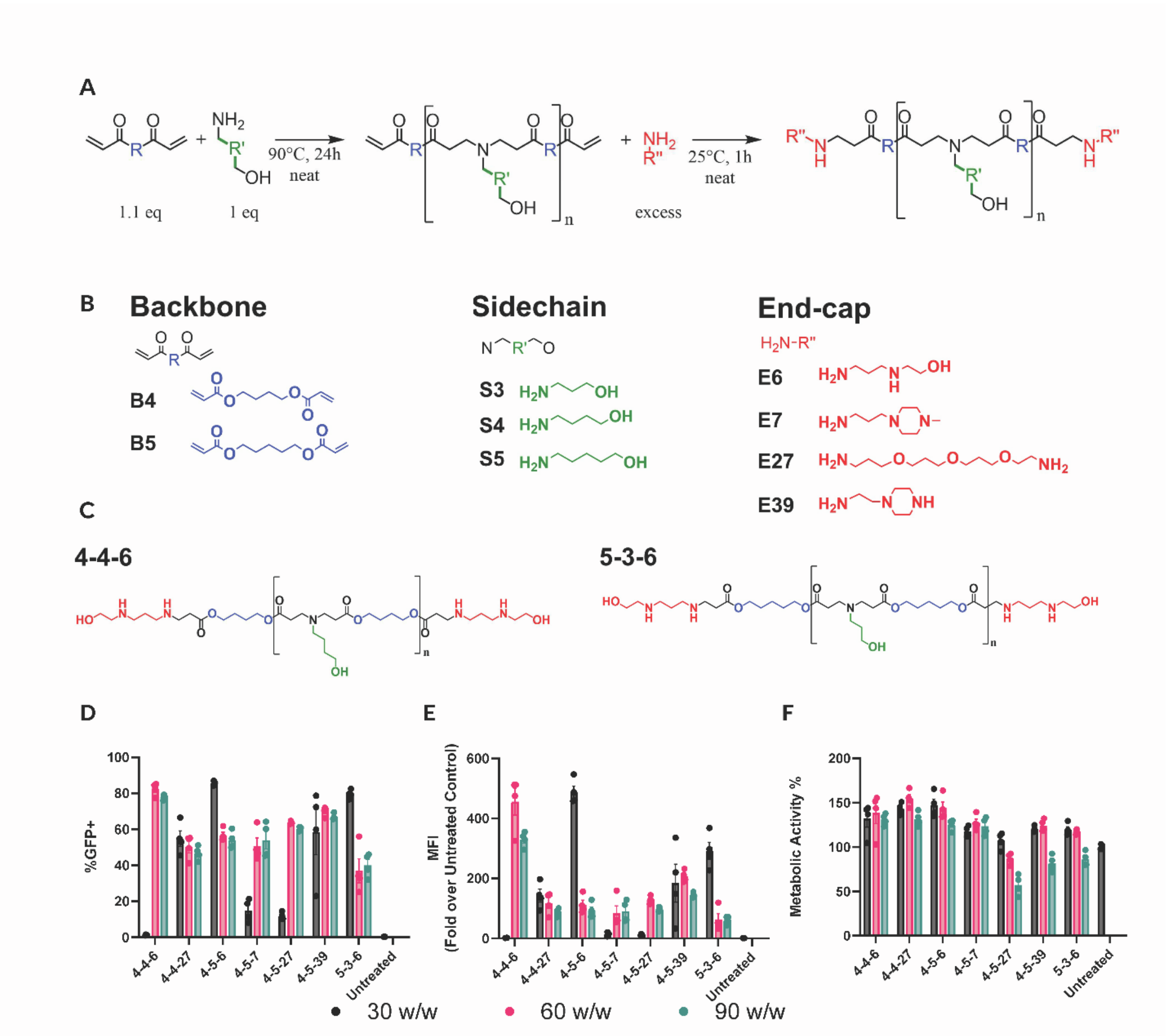

Figure S1: *In vitro* delivery efficiency for various PBAE polymers. (A) PBAE synthesis via Michael addition reaction. **(B)** PBAE monomer structures. **(C)** PBAE 4-4-6 and 5-3-6 structures. **(D)** Transfection of B16-F10 cells by seven PBAE polymers, as determined by flow cytometry. **(E)** Normalized GFP mean fluorescence intensity after transfection with PBAE NPs. **(F)** B16-F10 cell viability after transfection with PBAE polymers, normalized to untreated. Each data bar represents means ± standard error of the mean with four biological replicates.

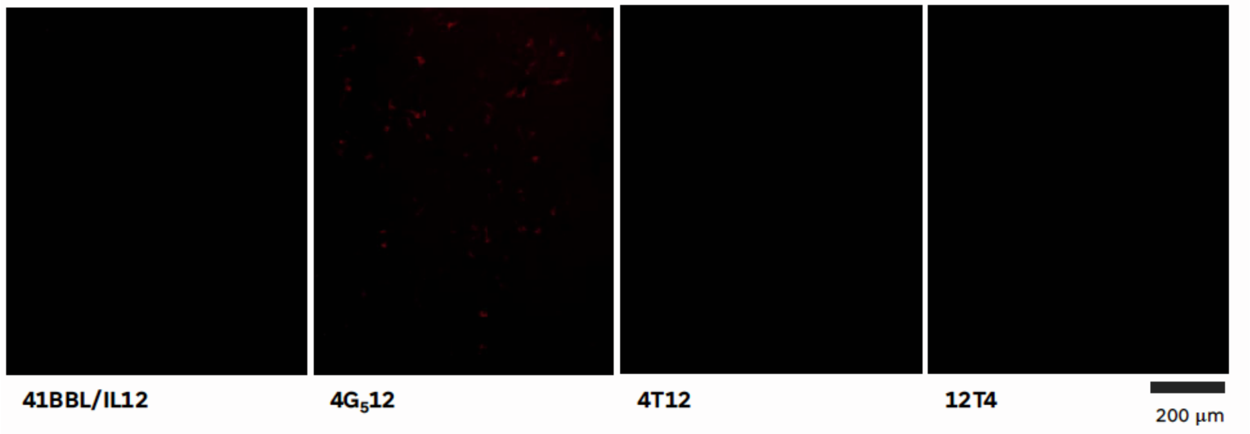

Figure S2. Microscopy images of B16-F10 cells *in vitro* transfected with 4-1BBL and IL-12 plasmids. Fluorescence imaging of B16-F10 cell surfaces for detection of IL-12. IL-12 presence on the cell surface is shown red.

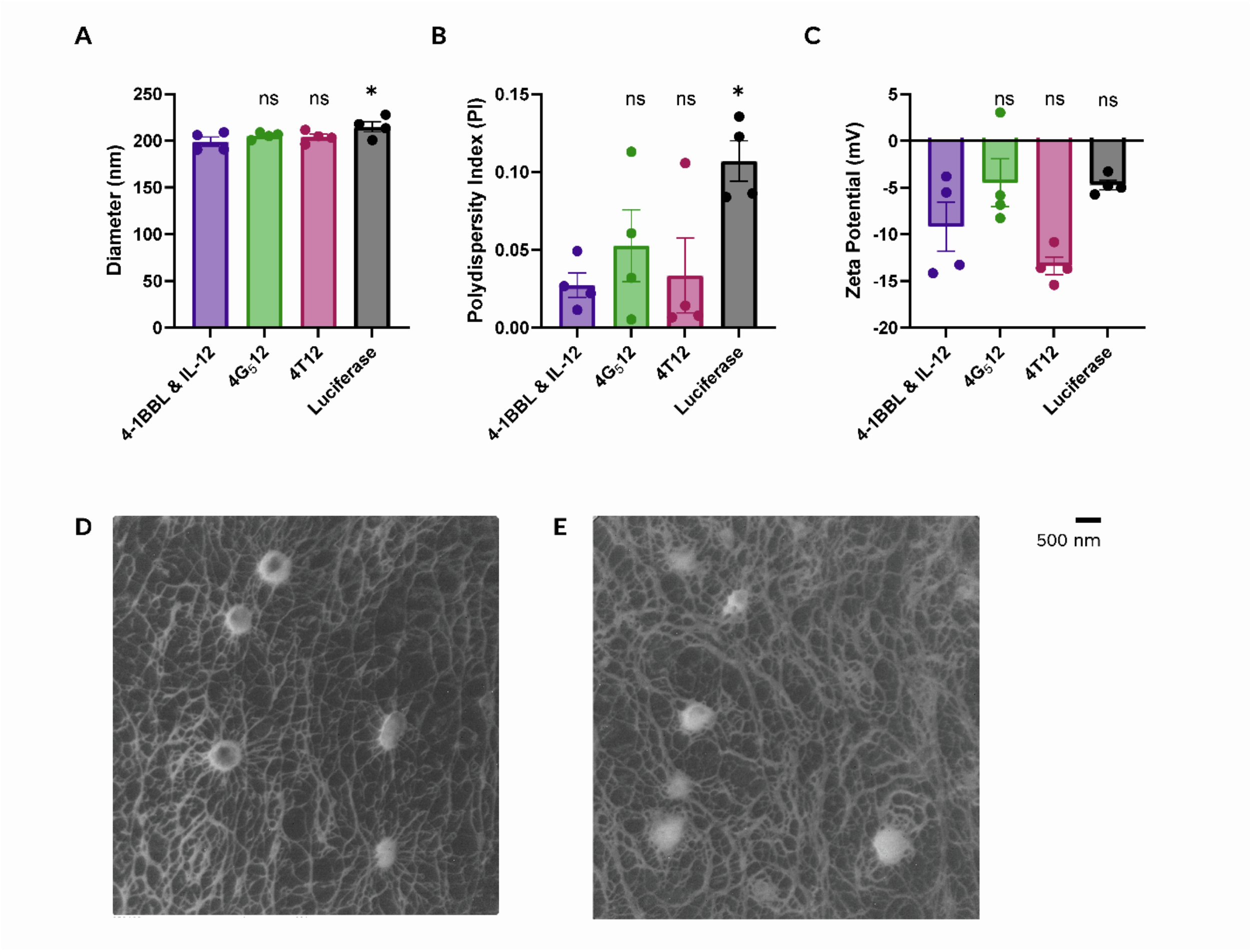

Figure S3. Characterization of nanoparticle formulations for *in vivo* delivery. (A) Nanoparticles were measured via DLS (One-way ANOVA, Dunnett's test, compared to 4-1BBL/IL-12). **(B)** Polydispersity indices are shown (One-way ANOVA, Dunnett's test, compared to 4-1BBL/IL-12). **(C)** Zeta potentials of nanoparticles shown (One-way ANOVA, Dunnett's test, compared to 4-1BBL/IL-12). **(D)** TEM imaging of dried NPs formed with two separate plasmids for 4-1BBL and IL-12. **(E)** TEM imaging of dried NPs formed with a bicistronic plasmid encoding both 4-1BBL and IL-12. Each data bar represents means ± standard error of the mean with four technical replicates. Significance is represented by *$p \leq 0.05$, **$p \leq 0.01$, ***$p \leq 0.001$, and ****$p \leq 0.0001$.

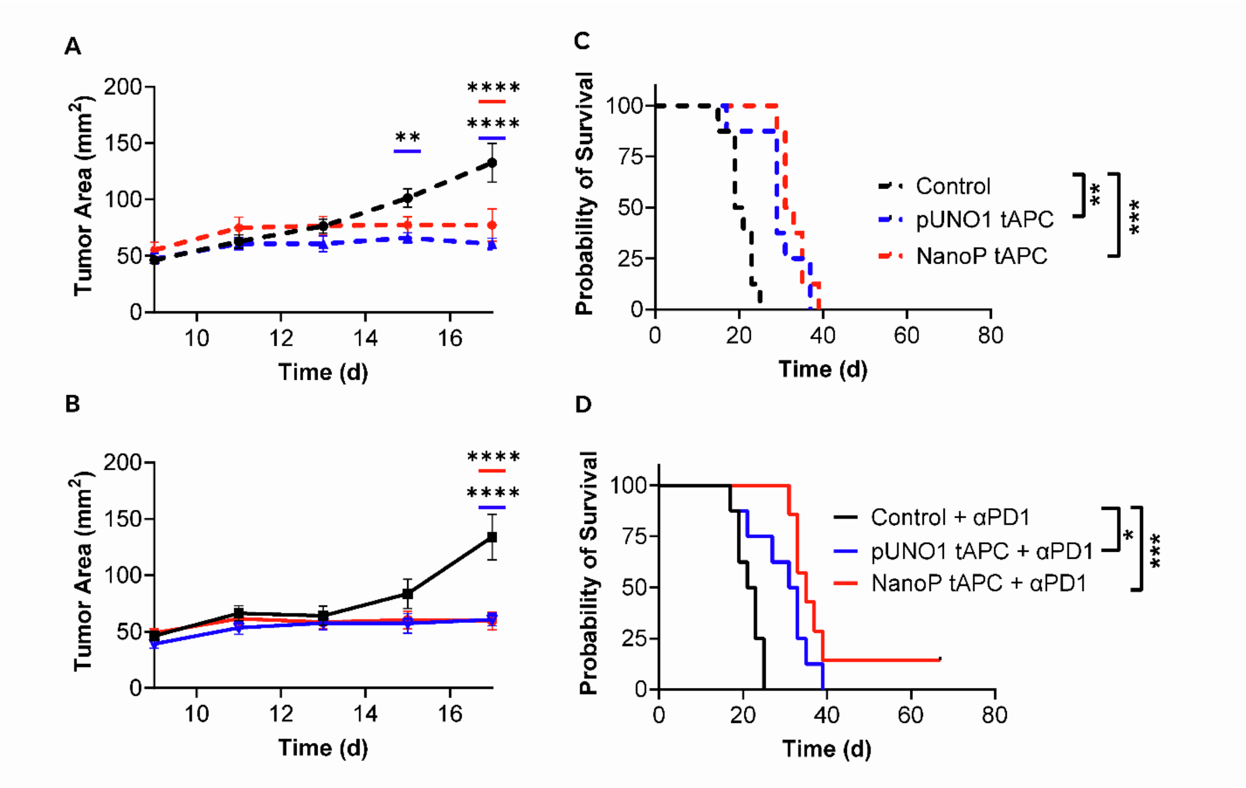

Figure S4. Survival in B16-F10 mouse model with antibiotic-gene free Nanoplasmids delivering 4-1BBL and IL-12 on separate plasmids. (A) Average tumor growth of tumors treated with tAPC NPs vs luciferase NPs (Two-way ANOVA, Dunnett's test, compared to control). **(B)** Average tumor growth of tumors treated with tAPC NPs + anti-PD1vs luciferase NPs + anti-PD1 (Two-way ANOVA, Dunnett's test, compared to control). **(C)** Survival curves of mice treated with tAPC NPs vs luciferase NPs. **(D)** Survival curves of mice treated with tAPC NPs + anti-PD1 vs luciferase NPs + anti-PD1. Each data bar represents mean ± SEM with four biological replicates. Significance is represented by *$p \leq 0.05$, **$p \leq 0.01$, ***$p \leq 0.001$, and ****$p \leq 0.0001$.

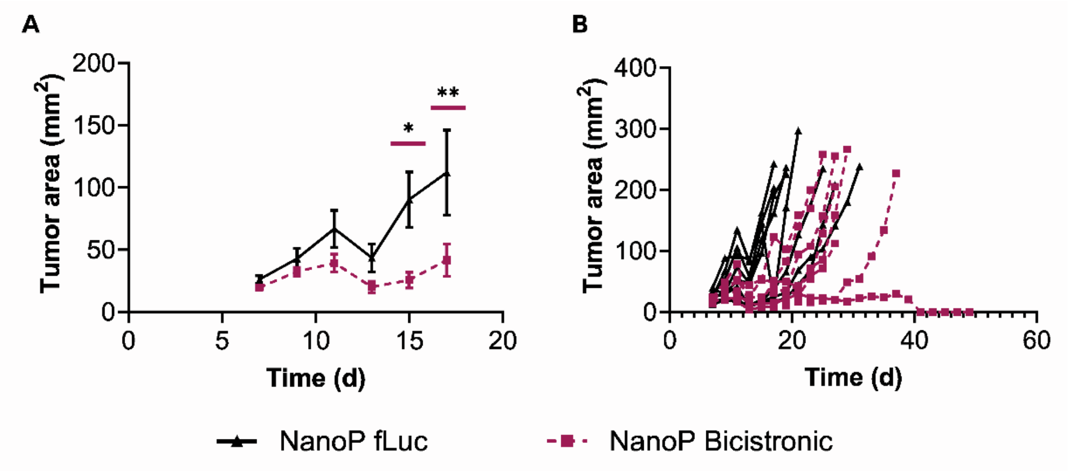

Figure S5. Tumor growth curves in B16-F10 mouse model comparing Nanoplasmid fLuc to Nanoplasmid 4T12. (A) Average tumor growth in mice treated with Nanoplasmid fLuc NPs + anti-PD1 vs Nanoplasmid 4T12 NPs + anti-PD1 (Two-way ANOVA, Sidak's test). **(B)** Individual tumor growth in mice treated with Nanoplasmid fLuc NPs + anti-PD1 vs Nanoplasmid 4T12 NPs + anti-PD1. Each data bar represents mean ± SEM with eight biological replicates. Significance is represented by *$p \leq 0.05$, **$p \leq 0.01$, ***$p \leq 0.001$, and ****$p \leq 0.0001$.

Table S1: Encoded amino acid sequences for 4-1BBL and IL-12 delivered. Amino acid sequences for the 4G$_5$12, 4T12, and 12T4 constructs are provided, with each component labeled below.

	IL-12 4-1BBL T2A **GS Linker** *Signal sequences are underlined*
**4G$_5$12**	MDQHTLDVEDTADARHPAGTSCPSDAALLRDTGLLADAALLSDTVRPTNAALPTDAAYPAV<br>NVRDREAAWPPALNFCSRHPKLYGLVALVLLLLIAACVPIFTRTEPRPALTITTSPNLGTRENN<br>ADQVTPVSHIGCPNTTQQGSPVFAKLLAKNQASLCNTTLNWHSQDGAGSSYLSQGLRYEE<br>DKKELVVDSPGLYYVFLELKLSPTFTNTGHKVQGWVSLVLQAKPQVDDFDNLALTVELFPCS<br>MENKLVDRSWSQLLLLKAGHRLSVGLRAYLHGAQDAYRDWELSYPNTTSFGLFLVKPDNP<br>WEGGGGSGGGGSGGGGSGGGGSGGGGSMWELEKDVYVVEVDWTPDAPGETVNLTCD<br>TPEEDDITWTSDQRHGVIGSGKTLTITVKEFLDAGQYTCHKGGETLSHSHLLLHKKENGIWS<br>TEILKNFKNKTFLKCEAPNYSGRFTCSWLVQRNMDLKFNIKSSSSSPDSRAVTCGMASLSAE<br>KVTLDQRDYEKYSVSCQEDVTCPTAEETLPIELALEARQQNKYENYSTSFFIRDIIKPDPPKNL<br>QMKPLKNSQVEVSWEYPDSWSTPHSYFSLKFFVRIQRKKEKMKETEEGCNQKGAFLVEKTS<br>TEVQCKGGNVCVQAQDRYYNSSCSKWACVPCRVRSVPGVGVPGVGRVIPVSGPARCLSQ<br>SRNLLKTTDDMVKTAREKLKHYSCTAEDIDHEDITRDQTSTLKTCLPLELHKNESCLATRETSS<br>TTRGSCLPPQKTSLMMTLCLGSIYEDLKMYQTEFQAINAALQNHNHQQIILDKGMLVAIDEL<br>MQSLNHNGETLRQKPPVGEADPYRVKMKLCILLHAFSTRVVTINRVMGYLSSA
**4T12**	MDQHTLDVEDTADARHPAGTSCPSDAALLRDTGLLADAALLSDTVRPTNAALPTDAAYPAV<br>NVRDREAAWPPALNFCSRHPKLYGLVALVLLLLIAACVPIFTRTEPRPALTITTSPNLGTRENN<br>ADQVTPVSHIGCPNTTQQGSPVFAKLLAKNQASLCNTTLNWHSQDGAGSSYLSQGLRYEE<br>DKKELVVDSPGLYYVFLELKLSPTFTNTGHKVQGWVSLVLQAKPQVDDFDNLALTVELFPCS<br>MENKLVDRSWSQLLLLKAGHRLSVGLRAYLHGAQDAYRDWELSYPNTTSFGLFLVKPDNP

	WEELSGSGEGRGSLLTCGDVEENPGPLRSPGRVNMCPQKLTISWFAIVLLVSPLMAMWELEKDVYVVEVDWTPDAPGETVNLTCDTPEEDDITWTSDQRHGVIGSGKTLTITVKEFLDAGQYTCHKGGETLSHSHLLLHKKENGIWSTEILKNFKNKTFLKCEAPNYSGRFTCSWLVQRNMDLKFNIKSSSSSPDSRAVTCGMASLSAEKVTLDQRDYEKYSVSCQEDVTCPTAEETLPIELALEARQQNKYENYSTSFFIRDIIKPDPPKNLQMKPLKNSQVEVSWEYPDSWSTPHSYFSLKFFVRIQRKKEKMKETEEGCNQKGAFLVEKTSTEVQCKGGNVCVQAQDRYYNSSCSKWACVPCRVRSVPGVGVPGVGRVIPVSGPARCLSQSRNLLKTTDDMVKTAREKLKHYSCTAEDIDHEDITRDQTSTLKTCLPLELHKNESCLATRETSSTTRGSCLPPQKTSLMMTLCLGSIYEDLKMYQTEFQAINAALQNHNHQQIILDKGMLVAIDELMQSLNHNGETLRQKPPVGEADPYRVKMKLCILLHAFSTRVVTINRVMGYLSSA
**12T4**	MCPQKLTISWFAIVLLVSPLMAMWELEKDVYVVEVDWTPDAPGETVNLTCDTPEEDDITWTSDQRHGVIGSGKTLTITVKEFLDAGQYTCHKGGETLSHSHLLLHKKENGIWSTEILKNFKNKTFLKCEAPNYSGRFTCSWLVQRNMDLKFNIKSSSSSPDSRAVTCGMASLSAEKVTLDQRDYEKYSVSCQEDVTCPTAEETLPIELALEARQQNKYENYSTSFFIRDIIKPDPPKNLQMKPLKNSQVEVSWEYPDSWSTPHSYFSLKFFVRIQRKKEKMKETEEGCNQKGAFLVEKTSTEVQCKGGNVCVQAQDRYYNSSCSKWACVPCRVRSVPGVGVPGVGRVIPVSGPARCLSQSRNLLKTTDDMVKTAREKLKHYSCTAEDIDHEDITRDQTSTLKTCLPLELHKNESCLATRETSSTTRGSCLPPQKTSLMMTLCLGSIYEDLKMYQTEFQAINAALQNHNHQQIILDKGMLVAIDELMQSLNHNGETLRQKPPVGEADPYRVKMKLCILLHAFSTRVVTINRVMGYLSSAGSGEGRGSLLTCGDVEENPGPLRSPVTMDQHTLDVEDTADARHPAGTSCPSDAALLRDTGLLADAALLSDTVRPTNAALPTDAAYPAVNVRDREAAWPPALNFCSRHPKLYGLVALVLLLLIAACVPIFTRTEPRPALTITTSPNLGTRENNADQVTPVSHIGCPNTTQQGSPVFAKLLAKNQASLCNTTLNWHSQDGAGSSYLSQGLRYEEDKKELVVDSPGLYYVFLELKLSPTFTNTGHKVQGWVSLVLQAKPQVDDFDNL

	ALTVELFPCSMENKLVDRSWSQLLLLKAGHRLSVGLRAYLHGAQDAYRDWELSYPNTTSFG LFLVKPDNPWE

Table S2: Detailed Statistical Analysis. Statistical testing for all of the figures and the corresponding statistical analysis are provided in Table S2. Please see the supplemental Table S2 excel file for the full details.
